# CARB-ES-19 Multicenter Study of Carbapenemase-Producing *Klebsiella pneumonia*e and *Escherichia coli* From All Spanish Provinces Reveals Interregional Spread of High-Risk Clones Such as ST307/OXA-48 and ST512/KPC-3

**DOI:** 10.3389/fmicb.2022.918362

**Published:** 2022-06-30

**Authors:** Javier E. Cañada-García, Zaira Moure, Pedro J. Sola-Campoy, Mercedes Delgado-Valverde, María E. Cano, Desirèe Gijón, Mónica González, Irene Gracia-Ahufinger, Nieves Larrosa, Xavier Mulet, Cristina Pitart, Alba Rivera, Germán Bou, Jorge Calvo, Rafael Cantón, Juan José González-López, Luis Martínez-Martínez, Ferran Navarro, Antonio Oliver, Zaira R. Palacios-Baena, Álvaro Pascual, Guillermo Ruiz-Carrascoso, Jordi Vila, Belén Aracil, María Pérez-Vázquez, Jesús Oteo-Iglesias, the GEMARA/GEIRAS-SEIMC/REIPI CARB-ES-19 Study Group, Mariela Martínez Ramírez

**Affiliations:** H. Universitario de Guadalajara, Guadalajara; Hospital Virgen de la Salud, Toledo; Hospital Virgen de la Concha Complejo Asistencial de Zamora, Zamora; Complejo Asistencial Universitario de Burgos, Burgos; H. General Universitario Gregorio Marañón, CIBERES Madrid; Hospital Universitario Insular Materno Infantil de Gran Canaria, Las Palmas; Hospital Universitario de Canarias, La Laguna, Sta. Cruz Tenerife; H. Universitario Río Hortega, Valladolid; Clínica Universidad de Navarra, Navarra; H. Universitario La Fe de Valencia, Valencia; H General Universitario de Ciudad Real, Ciudad Real; Hospital de Cabueñes, Asturias; Hospital San Pedro de Alcántara, Cáceres; Hospital Universitario Badajoz, Badajoz; Complejo Asistencial Universitario de León, León; Fundación Hospital de Calahorra Megalab, La Rioja; H. Verge de la Cinta de Tortosa, Tarragona; H. Universitario Miguel Servet, Zaragoza; H. San Jorge de Huesca, Huesca; H. San Pedro, La Rioja; H. Universitario Arnau de Vilanova, Lleida; Hospital Regional Universitario de Málaga, Málaga; Hospital Universitario Lucus Augusti, Lugo; Hospital Universitario Cruces, Vizcaya; H. General Universitario de Alicante, Alicante; Hospital General de Castellón, Castellón; Hospital Dr. Josep Trueta de Girona, Girona; H. Universitario 12 de Octubre, Madrid; Hospital Nuestra Señora de Sonsoles Ávila; Complejo Hospitalario de Salamanca, Salamanca; Real Hospital General de Segovia, Segovia; H. Clínico Universitario Lozano Blesa, Zaragoza; C. Hospitalario Universitario de Albacete, Albacete; Hospital General La Mancha Centro Alcazar, Ciudad Real; Hospital General Virgen de La Luz, Cuenca; Complejo Asistencial de Soria, Soria; Hospital Universitari Parc Taulí, Institut d’Investigació i Innovació Parc Taulí I3PT, Universitat Autònoma de Barcelona, Sabadell, Barcelona; H. Germans Trias I Pujol, Badalona Barcelona; Hospital Obispo Polanco, Teruel; H. General de Granollers, Barcelona; H. Virgen de la Victoria, Málaga; H. Clínico San Cecilio, Granada; Hospital Virgen de las Nieves, Granada; Jerez de la Frontera, Cádiz; H. Puerta del Mar, Cádiz; Hospital Universitario de Jaén, Jaén; Hospital de Poniente, Almería; Hospital Juan Ramón Jimenez Huelva; Complejo Hospitalario de Torrecárdenas, Almería; Complejo Hospitalario Universitario de Vigo, Pontevedra; Complejo Hospitalario de Orense, Orense; Hospital Santos Reyes, Aranda de Duero, Burgos; Hospital Clínico Universitario de Santiago de Compostela A Coruña; H. Universitario de Donostia, Guipuzcoa; Hospital Universitario de Basurto, IIS Biocruces, Bizkaia; H. General Río Carrión, Palencia; H. Universitario de Álava, Álava; H. General Universitario de Elche, Alicante; Hospital Virgen de la Arrixaca de Murcia, Murcia; Hospital Universitario de Bellvitge, Barcelona; H. Royo Villanova Zaragoza; H. del Mar, Barcelona; H. Virgen Macarena, CIBERINFEC, Seville; ^1^Laboratorio de Referencia e Investigación en Resistencia a Antibióticos e Infecciones Relacionadas con la Asistencia Sanitaria, Centro Nacional de Microbiología, Instituto de Salud Carlos III, Madrid, Spain; ^2^Escuela Internacional de Doctorado, Ciencias Biomédicas y Salud Pública - IMIENS (UNED), Madrid, Spain; ^3^Unidad de Enfermedades Infecciosas y Microbiología, Hospital Universitario Virgen Macarena, Instituto de Biomedicina de Sevilla (Hospital Universitario Virgen Macarena/CSIC/Universidad de Sevilla), Seville, Spain; ^4^CIBER de Enfermedades Infecciosas (CIBERINFEC), REIPI, Instituto de Salud Carlos III, Madrid, Spain; ^5^Servicio de Microbiología, Hospital Universitario Marqués de Valdecilla, IDIVAL, Santander, Spain; ^6^Servicio de Microbiología, Hospital Universitario Ramón y Cajal, Instituto Ramón y Cajal de Investigación Sanitaria (IRYCIS), Madrid, Spain; ^7^Servicio Microbiología, Hospital Universitario A Coruña, Instituto Investigación Biomédica A Coruña (INIBIC), A Coruña, Spain; ^8^Microbiology Unit, Reina Sofia University Hospital, Maimonides Biomedical Research Institute of Cordoba (IMIBIC), Córdoba, Spain; ^9^ Departament de Genetica i Microbiologia, Servei de Microbiologia, Hospital Universitari Vall d’Hebron, Universitat Autònoma de Barcelona, Barcelona, Spain; ^10^Servicio de Microbiología, Hospital Universitario Son Espases, Instituto de investigación sanitaria Illes Balears (IdISBa), Palma de Mallorca, Spain; ^11^Servicio de Microbiología, Hospital Clínic de Barcelona, ISGlobal Barcelona Institute for Global Health, Barcelona, Spain; ^12^Microbiology Department, Hospital de la Santa Creu i Sant Pau, Universitat Autónoma de Barcelona (UAB), Sant Pau Biomedical Research Institute (IIB Sant Pau), Barcelona, Spain; ^13^Department of Agricultural Chemistry, Soil Science and Microbiology, University of Córdoba, Córdoba, Spain; ^14^Departamento de Microbiología, Universidad de Sevilla, Seville, Spain; ^15^Servicio de Microbiología Clínica, Hospital Universitario La Paz (IdiPAz), Madrid, Spain

**Keywords:** CARB-ES-19 study, carbapenemases, whole genome sequencing, *Klebsiella pneumoniae*, high-risk clones

## Abstract

**Objectives:**

CARB-ES-19 is a comprehensive, multicenter, nationwide study integrating whole-genome sequencing (WGS) in the surveillance of carbapenemase-producing *K. pneumoniae* (CP-Kpn) and *E. coli* (CP-Eco) to determine their incidence, geographical distribution, phylogeny, and resistance mechanisms in Spain.

**Methods:**

In total, 71 hospitals, representing all 50 Spanish provinces, collected the first 10 isolates per hospital (February to May 2019); CPE isolates were first identified according to EUCAST (meropenem MIC > 0.12 mg/L with immunochromatography, colorimetric tests, carbapenem inactivation, or carbapenem hydrolysis with MALDI-TOF). Prevalence and incidence were calculated according to population denominators. Antibiotic susceptibility testing was performed using the microdilution method (EUCAST). All 403 isolates collected were sequenced for high-resolution single-nucleotide polymorphism (SNP) typing, core genome multilocus sequence typing (cgMLST), and resistome analysis.

**Results:**

In total, 377 (93.5%) CP-Kpn and 26 (6.5%) CP-Eco isolates were collected from 62 (87.3%) hospitals in 46 (92%) provinces. CP-Kpn was more prevalent in the blood (5.8%, 50/853) than in the urine (1.4%, 201/14,464). The cumulative incidence for both CP-Kpn and CP-Eco was 0.05 per 100 admitted patients. The main carbapenemase genes identified in CP-Kpn were *bla*_OXA–48_ (263/377), *bla*_KPC–3_ (62/377), *bla*_VIM–1_ (28/377), and *bla*_NDM–1_ (12/377). All isolates were susceptible to at least two antibiotics. Interregional dissemination of eight high-risk CP-Kpn clones was detected, mainly ST307/OXA-48 (16.4%), ST11/OXA-48 (16.4%), and ST512-ST258/KPC (13.8%). ST512/KPC and ST15/OXA-48 were the most frequent bacteremia-causative clones. The average number of acquired resistance genes was higher in CP-Kpn (7.9) than in CP-Eco (5.5).

**Conclusion:**

This study serves as a first step toward WGS integration in the surveillance of carbapenemase-producing Enterobacterales in Spain. We detected important epidemiological changes, including increased CP-Kpn and CP-Eco prevalence and incidence compared to previous studies, wide interregional dissemination, and increased dissemination of high-risk clones, such as ST307/OXA-48 and ST512/KPC-3.

## Introduction

The rapid spread of carbapenemase-producing Enterobacterales (CPE) is a threat to individual and public health worldwide; infections caused by CPE significantly increase morbidity and mortality (Barrasa-Villar et al., 2017). The World Health Organization has included CPE as a critical priority issue (Tacconelli et al., 2018). The carbapenemases most frequently found in Enterobacterales are KPC, OXA-48, VIM, IMP, and NDM, although prevalence rates differ according to the geographical area considered (Grundmann et al., 2017).

Previous studies performed in Spain revealed the rapid evolution of CPEs from isolated cases in 2009 (Miró et al., 2013) to interregional dissemination in 2013 (Oteo et al., 2015). Recent European studies have indicated epidemiological changes in CPE infections (Haller et al., 2019; Ludden et al., 2020; Oteo-Iglesias et al., 2020; Di Pilato et al., 2021). According to the first structured survey on the occurrence of carbapenemase-producing *K. pneumoniae* and *E. coli* in European hospitals (Grundmann et al., 2017), Spain had the fourth highest incidence in Europe (0.04 cases per 100 patients) after Italy, Greece, and Montenegro.

However, recent multicenter studies with adequate geographical representation are scarce, since regions with low prevalence are often underrepresented.

Accurate data collection at the national level is required for the successful implementation of CPE control measures.

The CARB-ES-19 project utilized the national antibiotic-resistance surveillance framework and was designed to provide continuity with previous national, multicenter studies (Miró et al., 2013; Oteo et al., 2015). This large-scale, nationwide, structured survey integrated whole-genome sequencing (WGS) analyses of two CPE species with high clinical and epidemiological impacts, *Klebsiella pneumoniae* (CP-Kpn) and *Escherichia coli* (CP-Eco), to (i) determine the prevalence and incidence of these microorganisms, (ii) describe their inter-regional distribution and molecular epidemiology, and (iii) describe their resistance mechanisms and susceptibility profiles.

## Materials and Methods

### Study Design and Isolates

CARB-ES-19 is a prospective, multicenter study designed to identify clinical cases associated with CP-Kpn and CP-Eco. In total, 71 hospitals, representing all 50 Spanish provinces, collected the first 10 non-duplicate consecutive isolates of carbapenem non-susceptible CP-Eco or CP-Kpn isolated from clinical samples from individual consecutive patients between February and May 2019. The geographical distribution of participating hospitals is available in the free and interactive online access tool Microreact.^1^

Not all participating hospitals had 10 CP-Eco or CP-Kpn isolates during the study period. Isolates from rectal exudates for the detection of EPC carriers were not included. All provinces (NUTS-3 regions in Spain) were represented by at least one hospital; seven of the provinces with the largest population were represented by more than one hospital (range 2–6). A similar study design was used by the European Centre for Disease Prevention and Control (ECDC) in the European survey of carbapenemase-producing Enterobacteriaceae (EuSCAPE; Grundmann et al., 2017).

Initial assays were performed at each participating hospital using standard microbiological methods. CPE isolates were identified according to the European Committee on Antimicrobial Susceptibility Testing (EUCAST) established meropenem cutoff value for CPE (meropenem MIC > 0.12 mg/L; European Society of Clinical Microbiology and Infectious Diseases [EUCAST], 2017). Confirmation of carbapenemase production was verified using at least one EUCAST-recommended method (European Society of Clinical Microbiology and Infectious Diseases [EUCAST], 2017), such as immunochromatography, biochemical (colorimetric) tests, carbapenem inactivation, or detection of carbapenem hydrolysis with MALDI-TOF.

In total, 10 hospitals and the Spanish National Centre of Microbiology (CNM) acted as node centers, performing molecular confirmation of standard carbapenemase genes using PCR (Supplementary Table 1). Confirmed CPE isolates were submitted to the Antibiotic Reference Laboratory of the CNM for WGS. Prevalence was estimated as the proportion of CP-Kpn and CP-Eco isolates relative to the total collected clinical *K. pneumoniae* and *E. coli* isolates, respectively. Overall cumulative incidence and incidence density estimates are reported as the number of admitted patients diagnosed with either CP-Kpn or CP-Eco per 100 admitted patients and per 1,000 patient-days, respectively. The denominators for the incidence and prevalence estimates were adjusted to the date on which the last isolate included in the study was collected in each hospital.

### Drug Susceptibility Testing

Antibiotic susceptibility testing was performed using the broth microdilution susceptibility method (DKYMGN SensititreTM panels, Thermo Fisher Scientific, United States) (International Organization for Standardization [ISO], 2006). Antibiotic gradient strips were used to study susceptibility to meropenem/vaborbactam and cefepime (bioMérieux, Marcy-l’Étoile, France) and to imipenem/relebactam, plazomicin, and cefiderocol (Liofilchem, Roseto degli Abruzzi, Italy) in Mueller Hinton agar (bioMérieux, Marcy-l’Étoile, France). EUCAST v12.0 clinical breakpoints and guidelines for Enterobacterales were used for interpretation. An FDA-approved susceptibility breakpoint of ≤ 2 mg/L was used for plazomicin.

### Genomic Library Preparation and Sequence Analysis

Genomic DNA paired-end libraries were generated using the Nextera XT DNA Sample Preparation Kit (Illumina Inc., San Diego, CA, United States). These libraries were sequenced using the Illumina HiSeq 500 next-generation sequencer with 2 × 150 bp paired-end reads (Illumina Inc.) Raw sequence data were submitted to the European Nucleotide Archive (PRJEB50822). The quality of short reads was assessed using FASTQC, and assembly into contigs was performed with Unicycler 0.4.8 (Wick et al., 2017). The quality of the assembly was assessed with QUAST.^2^ Prokka v1.14-beta (Seemann, 2014) was used for automatic *de novo* assembly annotation.

### Phylogenetic Analysis

Assembly contigs were used as input for Roary version 3.13.0 (Page et al., 2015). An alignment of 2,645 core genes (present in > 99% of isolates), comprising 2,415,034 bp, was generated for *K. pneumoniae*. Variable positions were extracted (85,696 single-nucleotide polymorphisms [SNPs]), and a maximum-likelihood phylogenetic tree of SNPs was constructed using RAxML version 7.0.4 (Stamatakis, 2006) with a general time-reversible model and gamma correction for site variation. The phylogenetic tree and associated metadata were visualized using Microreact and iTOL.^3^

Sequence types (STs) were calculated according to multilocus sequence typing (MLST) schemes of the Institut Pasteur and the University of Warwick for *K. pneumoniae* and *E. coli*, respectively, using Ariba version 2.6.2 (Hunt et al., 2017). A simple diversity index (SDI; Gastmeier et al., 2006) was applied to analyze population diversity. Core genome MLST (cgMLST) was performed, consisting of 2,538 *K. pneumoniae* targets provided by SeqSphere + version 3.5.0 (Ridom, Münsten, Germany).

### Analysis of Antimicrobial Resistance, Virulence Genes, and Capsular Locus

Antibiotic resistance genes were analyzed by SRST2 (Inouye et al., 2014) using the ARG-ANNOT database (Gupta et al., 2014) and ResFinder (CGE server^4^), with ID thresholds of 100% for β-lactamase variants and 98% for other genes. The K-locus and virulence genes were characterized using Kleborate.^5^ The presence of *ybt*, *clb*, and *iuc* was used to assign a virulence score, as described by Lam et al. (2021).

### Characterization of Plasmids Carrying Carbapenemase Genes

The plasmids carrying the carbapenemase genes in six representative *K. pneumoniae* isolates (ST307/KPC-3, ST512/KPC-3, ST512/KPC-23, ST147/NDM-1, ST307/OXA-48, and ST11/VIM-1) were reconstructed by the in-house script (PlasmidID^6^). PlasmidID was used to (i) map reads over a plasmid curated database to find those with the higher coverage and *de novo* assembly of these reads and (ii) make local alignments to localize resistance and replicative genes (Pérez-Vázquez et al., 2019a).

### Ethics Statement

This study was authorized by the Spanish Agency for Medicines and Health Products (code JOI-AVI-2019-01). The Research Ethics Committee of the University Hospitals Virgen Macarena and Virgen del Rocio (Sevilla, Spain) approved this study.

## Results

### Bacterial Isolates, Prevalence, Incidence, and Carbapenemase Types

Of 403 CPE cases, 377 (93.5%) were identified as CP-Kpn and 26 (6.5%) as CP-Eco. Patients were mainly men (211, 52.4%) older than 65 years (280, 69.5%). CPE isolates were collected from urine (215, 53.3%), wounds and abscesses (61, 15.1%), blood (52, 12.9%), respiratory samples (47, 11.7%), and other locations (28, 6.9%).

**FIGURE 1:**
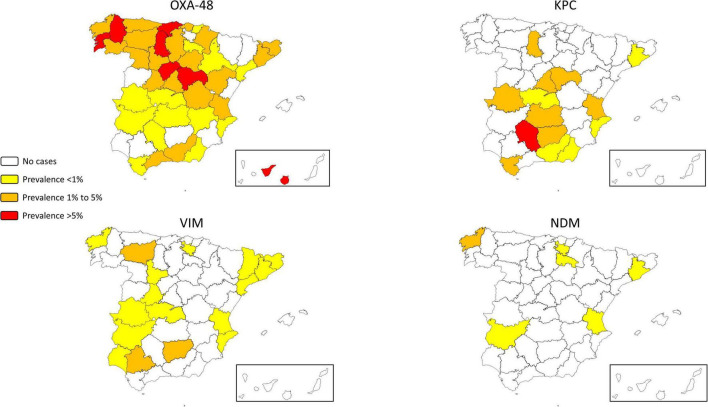
Geographical distribution of the prevalence of carbapenemase-producing *Klebsiella pneumoniae* according to carbapenemase groups.

At least one case was identified in 62 (87.3%) of participating hospitals and in 46 (92%) of 50 Spanish provinces. Participating hospitals isolated a total of 15,100 and 70,760 isolates of *K. pneumoniae* and *E. coli*, respectively. The average CP-Kpn prevalence was 2.5% (377/15100; interprovincial range: 0–17.3%), with 16 (22.5%) hospitals reporting prevalences greater than 5%. The average prevalence of CP-Eco was 0.04% (26/70,760; interprovincial range: 0–0.5%; Supplementary Table 2). The prevalence distribution by province is detailed in Figure 1 and Supplementary Table 2. CP isolates were more prevalent in blood (CP-Kpn: 5.8%, 50/853; CP-Eco: 0.06%, 2/3,353) than in urine (CP-Kpn: 1.4%, 201/14,464; CP-Eco: 0.02%, 14/56,848).

Overall, cumulative incidence and incidence density estimates were 0.05 per 100 admitted patients (interprovincial range: 0–0.34) and 0.08 per 1,000 patient-days (interprovincial range: 0–0.58), respectively (Supplementary Table 2).

The carbapenemase genes detected in CP-Kpn were *bla*_OXA–48_ (263 isolates, 69.8%), *bla*_KPC–3_ (62, 16.4%), *bla*_VIM–1_ (28, 7.4%), *bla*_NDM–1_ (12, 3.2%), *bla*_KPC–23_ (7, 1.9%), *bla*_KPC–2_ (5, 1.3%), *bla*_OXA–245_ (3, 0.8%), with *bla*_IMP–8_, *bla*_GES–2_, *bla*_NDM–3_, *bla*_NDM–23_, and *bla*_OXA–505_ in one isolate each (0.3%). In CP-Eco, *bla*_OXA–48_ (19, 73.1%) was predominant, followed by *bla*_VIM–1_ (5, 19.2%), *bla*_KPC–3_ (2, 7.7%), and *bla*_NDM–5_ (1, 3.8%). Eight CP-Kpn (two OXA-48 + VIM-1, two OXA-48 + KPC, and one each of OXA-48 + NDM-1, VIM-1 + NDM-1, NDM-1 + GES-2, and VIM-1 + NDM-3) and one CP-Eco (OXA-48 + VIM-1) isolates co-produced two carbapenemase types.

The Canary Islands and northern Spain had more OXA-48-producing CP-Kpn, whereas southern Spain had more KPC-producing CP-Kpn (Figure 1, Supplementary Table 2).

### Antibiotic Susceptibility Testing

The antibiotic susceptibilities of CP-Kpn isolates are listed in Tables 1 and 2. The overall rates of susceptibility to carbapenems were 54.6% for imipenem, 52.3% for meropenem, and 3.4% for ertapenem (Table 1A), with all isolates non-susceptible to at least one carbapenem.

**TABLE 1:** Antibiotic susceptibility of 377 carbapenemase-producing *Klebsiella pneumoniae* isolates as determined by the microdilution method and antibiotic gradient strips (antibiotics with *) according to EUCAST clinical breakpoints: (A) General results. (B) Results according to carbapenemase groups.

A					

Antibiotics	S (%)	R (%)	MIC_50_^a^	MIC_90_^a^	Range^a^
Cefiderocol*	93.9	6.1	0.12	1	≤0.015–16
Plazomicin*	93.4	6.1	1	2	0.25 to >256
Colistin	90.5	9.5	1	2	0.5 to >8
Meropenem/vaborbactam*	89.4	10.6	0.5	16	≤0.015 to >128
Ceftazidime/avibactam	84.1	15.9	2	>16	≤0.5 to >16
Imipenem/relebactam*	77.4	22	1	8	0.12 to >32
Amikacin	72.9	27.1	8	>32	≤4 to >32
Imipenem	54.6	36.9	2	>16	≤0.5 to >16
Meropenem	52.3	28.4	2	>16	0.25 to >16
Gentamicin	45.6	54.4	4	>8	≤0.5 to >8
Trimethoprim/sulfamethoxazole	26.8	71.1	>8	>8	≤1 to >8
Tobramycin	25.7	74.3	>8	>8	≤1 to >8
Aztreonam	14.9	83.3	>32	>32	≤0.5 to >32
Cefepime*	11.9	84.1	32	>256	0.12 to >256
Ceftazidime	10.6	85.9	>16	>16	≤0.5 to >16
Ceftolozane/tazobactam	9.8	90.2	>32	>32	≤0.5 to >32
Cefotaxime	6.1	90.5	>8	>8	≤0.5 to >8
Ciprofloxacin	5.6	94.2	>2	>2	≤0.06 to >2
Ertapenem	3.4	96.6	>2	>2	0.25 to >2

**B**					

**Antibiotic**	**Susceptibility (%)**					
	**OXA-48-group-producing isolates (*n* = 262)**		**KPC-group-producing isolates (*n* = 72)**		**MBL-group-producing isolates (*n* = 37)**

Cefiderocol*	95.8		86.1		94.6
Plazomicin*	93.5		98.6		86.5
Colistin	92.4		81.9		91.9
Meropenem/vaborbactam*	89.3		100		73
Ceftazidime/avibactam	95.4		90.3		0
Imipenem/relebactam*	75.2		100		56.7
Amikacin	86.6		33.3		56.8
Imipenem	68.3		13.9		43.2
Meropenem	64.9		13.9		45.9
Gentamicin	47.3		48.6		32.4
Trimethoprim/sulfamethoxazole	34.7		6.9		13.5
Tobramycin	32.8		9.7		8.1
Aztreonam	17.6		0		27
Cefepime*	16.8		0		0
Ceftazidime	15.3		0		0
Ceftolozane/tazobactam	14.1		0		0
Cefotaxime	8.4		0		0
Ciprofloxacin	7.6		1.4		0
Ertapenem	2.3		0		16.2

*S, susceptible, standard dosing regimen; R, resistant; MIC, minimum inhibitory concentrations.*

*^a^Expressed in mg/L.*

*Isolates with two carbapenemases of different groups are excluded.*

**TABLE 2:** High-risk carbapenemase-producing *Klebsiella pneumoniae* clones defined according to combinations of sequence type/carbapenemase.

High-risk clones (*n*)	Hospitals (*n*)	Geographical distribution*	Evolution trends (%) 2013→2019**	Representation in bacteremia (%)	Carbapenemase genes (%)	Other prevalent acquired resistance genes (%)
ST307/OXA-48 (62)	23	Andalucía, Castilla La Mancha, Castilla y León, Extremadura, Cataluña, Galicia, Canarias, País Vasco, Comunidad Valenciana, Madrid	1.4→16.4	14	*bla*_OXA–48_ (100)	*bla*_SHV–28_ *(95.1), bla*_CTX–M–15_ (88.5), *aac(6’)-Ib-cr* (78.7), *qnrB1* (78.7), *ant(3’)-Ia* (75.4), *sul2* (73.8), *bla*_OXA–1_ (73.8), *aph(3”)-Ib* (73.8), *aph(6)-Id* (73.8), *catB3* (73.8%), *dfrA14* (73.8), *aac(3)-IIa* (72.1), *bla*_TEM–1b_ (70.5).
ST11/OXA-48 (62)	22	Andalucía, Castilla La Mancha, Castilla y León, Cantabria, Cataluña, La Rioja, Canarias, País Vasco, Madrid	24.1→16.4	12	*bla*_OXA–48_ (100),	*bla*_CTX–M–15_ (96.7), *bla*_SHV–182_ (95.1), *bla*_OXA–1_ (60.7), *aac(6’)-Ib-cr* (63.9), *catB*3 (63.9), *aac(3)-IIa* (55.7), *qnrB1*-like (57.4).
ST512-ST258/KPC (52)	10	Andalucía, Castilla La Mancha, Comunidad Valenciana, Cataluña, Madrid	0→13.8	24	*bla*_KPC–3_ (86.5) *bla*_KPC–23_ (13.5)	*bla*_SHV–182_ *(97.9), aph(3”)-Ia (95.8), bla*_OXA–9_ *(95.8), aac(6’)-Ib* (93.8), *ant(3’)-Ia* (93.8), *sul1* (93.8), *dfrA12* (93.3), *catA1* (91.7), *dfrA12* (91.7), *mphA* (91.7), *bla_TEM–187_* (64.6).
ST15/OXA-48 like (43)	16	Andalucía, Castilla La Mancha, Madrid, Aragón, Cataluña, Canarias, País Vasco, Galicia	8.2→11.4	16	*bla*_OXA–48_ (93) *bla*_OXA–245_ (7)	*bla*_SHV–28_ *(100), ant(3’)-Ia (83.7), bla*_OXA–1_ (74.4%), *bla*_CTX–M–15_ (72.1), *aac(6’)-Ib-cr* (76.7), *catB*3 (74.4), *dfrA14* (74.4), *aph(3”)-Ib* (60.5), *aph(6)-Id* (58.1), *sul2* (58.1), *bla*_TEM–1b_ (53.5).
ST147/OXA-48 (22)	10	Andalucía, Cataluña, Galicia, País Vasco, Asturias, Navarra	2.5→5.8	0	*bla*_OXA–48_ (95.5) *bla*_OXA–505_(4.5)	*ant(3’)-Ia (90.9), bla*_CTX–M–15_ (86.4), *bla*_SHV–67_ (86.4), *aac(6’)-Ib* (72.7), *AAR-2* (72.7), *rmtF* (63.6), *qnrB1* (47.6), *dfrA14* (68.2), *mphA* (63.6), *dfrA12* (59.1), *sul1* (59.1).
ST307/KPC-3 (15)	4	Madrid, Extremadura, Castilla La Mancha	0→4	4	*bla*_KPC–3_ (100)	*bla*_CTX–M–15_ (100), *bla_OXA–1_* (100), *bla_OXA–9_* (100), *aac(6’)-Ib-cr* (100), *aac(3)-IIa* (100), *ant(3’)-Ia* (100), *aph(3”)-Ib* (100), *aph(6)-Id* (100), *catB3* (100), *dfrA14* (100), *bla*_SHV–67_ (100), *sul2* (100), *qnrB1*-like (71.4).
ST392/OXA-48 (14)	11	Andalucía, Castilla La Mancha, Madrid, Cataluña, Comunidad Valenciana, Galicia	0→3.7	4	*bla*_OXA–48_ (100).	*bla*_SHV–67_ *(100), aph(3”)-Ib (92.9), aph(6)-Ib (92.9), sul2* (86.7), *bla*_TEM1–b_ (85.7), *bla_CTX–M–15_* (78.6), *dfrA14* (50), *qnrB1* (40), *aac(6’)-Ib-cr* (57.1), *bla_OXA–1_* (50), *catB3* (50).
ST147/NDM-1 (7)	4	Cataluña, Galicia	0→1.9	2	*bla*_NDM–1_ (100)	*aadA1(100), ARR(100), bla*_CTX–M–1–group_ (85.7), *bla*_SHV–67_ (100), *sul1* (100), *aac(6’)-Ib* (71.4), *dfrA12* (71.4), *dfrA14* (71.4), *qnrB1* (71.4), *rmtF*1 (71.4), *aph(3”)-lb* (57.1), *aph*(6)-ld (57.1).

*Autonomous Communities in which the high-risk clones were detected.

**Evolution trends detected based on the results of a previous Spanish study conducted in 2013 (Oteo et al., 2015) relative to the study in 2019.

The most active antibiotics *in vitro* were cefiderocol (93.9% susceptibility), plazomicin (93.4%), colistin (90.5%), meropenem/vaborbactam (89.4%), ceftazidime/avibactam (84.1%), and imipenem/relebactam (78%; Table 1A). However, these numbers varied significantly depending on the carbapenemase type (Table 1B).

In general, CP-Eco isolates were more susceptible to antibiotics (Supplementary Table 3) than CP-Kpn isolates, with the greatest differences observed for tigecycline (84.6% in CP-Eco vs. 29.2% in CP-Kpn).

### High-Risk Clones of Carbapenemase-Producing *Klebsiella pneumoniae*

CP-Kpn isolates were grouped into 48 STs (SDI: 12.7; mean: 7.8 isolates per ST; range: 1–82). A new ST was detected in an OXA-48-producing *K. pneumoniae* strain. The most prevalent STs (≥10 isolates) were ST307 (82, 21.7%), ST11 (68, 18%), ST258/512 complex (4/48, 13.8%), ST15 (48, 12.7%), ST147 (36, 9.5%), and ST392 (15, 4%), accounting for 79.7% of all CP-Kpn isolates. ST307 and ST11 expressed four different carbapenemase types (OXA-48, KPC, VIM-1, and NDM-1), but ST258/512 only expressed KPC. Among CP-Eco isolates, 21 different STs were identified (SDI: 80.8; mean: 1.2 isolates per ST; range: 1–4), with only ST131 (4 isolates, 15.4%) expressing more than two carbapenemases.

Considering ST/carbapenemase combinations, eight high-risk CP-Kpn clones (≥ 7 isolates) were detected, with ST307/OXA-48 (16.4%), ST11/OXA-48 (16.4%), ST512-ST258/KPC (13.8%), and ST15/OXA-48-like (11.4%) as the most common combinations (Table 2). These high-risk clones were detected in at least four hospitals and two autonomous communities, suggesting interregional dissemination (Table 2). ST512-258/KPC and ST15/OXA-48-like were the most frequent bacteremia-producing clones, responsible for 24 and 16% of CP-Kpn–induced bacteremia, respectively (Table 2).

### Phylogenetic Analysis of Carbapenemase-Producing *Klebsiella pneumoniae*

**FIGURE 2:**
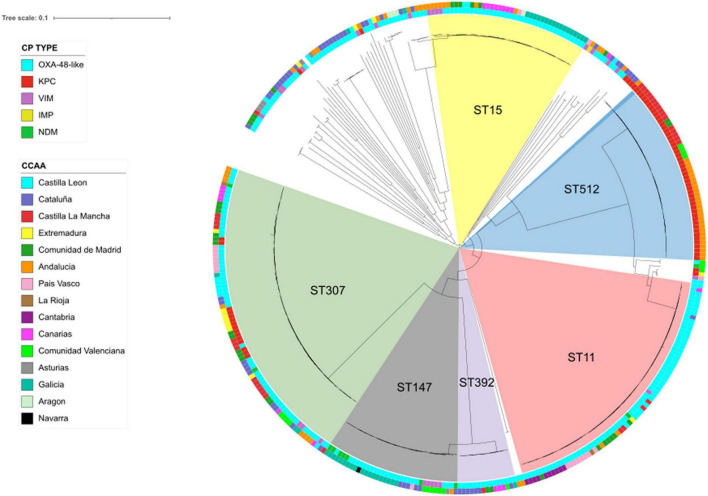
Population structure of carbapenemase-producing *Klebsiella pneumoniae* isolates: maximum-likelihood tree showing the relationship between isolates, with more frequent STs displayed in the inner ring and labeled. The inner ring colors indicate the carbapenemase type (CP type); the outer ring colors indicate geographical distribution (CCAA: autonomous communities); and branch lengths are indicative of the number of SNPs.

A total of 92,608 high-quality SNPs, identified by referencing the sequence of *K. pneumoniae* strain NTUH-K2044, were used to construct a maximum-likelihood phylogenetic tree (Figure 2). The median pairwise distance between isolates was 9,678 SNPs (range: 0–13,254 SNPs). CP-Kpn isolates were grouped into six major clusters (clusters 1–6; Figure 2). The main characteristics of these six clusters are detailed in Supplementary Table 4. The average difference between isolates from different clusters was 10,580 SNPs.

**FIGURE 3:**
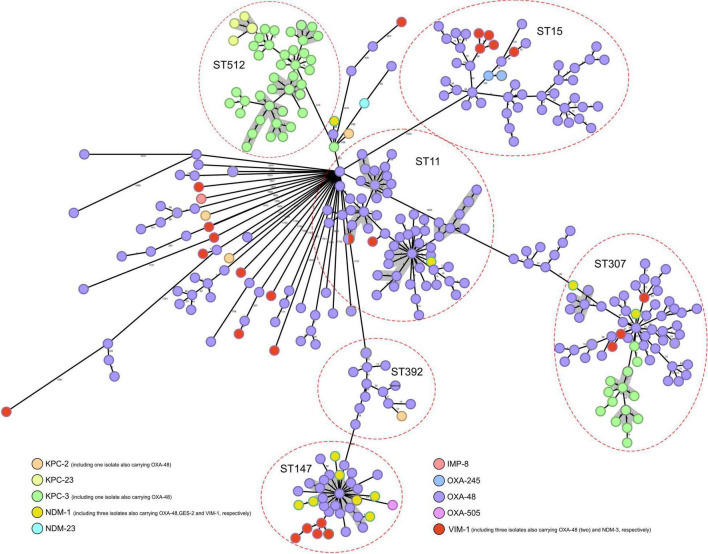
Population structure of carbapenemase-producing *Klebsiella pneumoniae*: minimum-spanning tree. Distances shown are based on cgMLST of 2,358 genes using the parameter “pairwise ignoring missing values.” Colors in each circle indicate carbapenemase type. Red ovals represent clusters. Circles can correspond to more than one isolate, indicating that they are identical. Gray shadows represent clusters of strains, applying a threshold of both 5 SNPs and 10 alleles.

Genome assemblies of all CP-Kpn isolates, analyzed using the gene-by-gene approach and allelic distance from cgMLST, are reflected in a minimum-spanning tree (Figure 3). The average allelic distances in pairwise comparisons of isolates were 45 alleles in cluster 1 (range: 0–235 alleles), 68 alleles in cluster 2 (range: 0–49), 64 alleles in cluster 3 (range: 0–139), 21 alleles in cluster 4 (range: 0–43), 30 alleles in cluster 5 (range: 0–106), and 39 alleles in cluster 6 (range: 0–64).

Applying a threshold of both 5 alleles and 10 SNPs, 12 groups with more than four closely related isolates were detected, with ST512 (4), ST11 (4), and ST307 (3) commonly identified (Figure 3).

### Identification and Distribution of Resistance and Virulence Genes

An average of 7.9 acquired resistance genes (ARGs) were detected in CP-Kpn isolates (range: 1–20 ARGs), and 5.5 ARGs were detected in CP-Eco isolates (range: 1–18 ARGs). The most frequent extended-spectrum β-lactamase (ESBL) gene was *bla*_CTX–M–15_ (247 overall, 61.3%), detected in 240 CP-Kpn (63.7%) and 7 CP-Eco (26.9%). Other ESBL genes identified were *bla*_CTX–M–9_ (7, 1.7%), *bla*_SHV–12_ (4, 1%), *bla*_CTX–M–3_ (2, 0.5%), *bla*_CTX–M–88_ (2, 0.5%), and *bla*_CTX–M–65_ (1, 0.25%).

The predominant aminoglycoside resistance genes encoded N-acetyltransferases *aac(6’)-Ib-cr* (189, 46.9%), *aac(3)-IIa* (141, 35%), and *aac(6’)-Ib* (70, 17.4%). Acquired 16S rRNA methyltransferase *rmtF*1 was detected in 19 CP-Kpn (5%) isolates, all belonging to ST147 and encoding *bla*_OXA–48_ (14) or *bla*_NDM–1_ (5).

ARGs encoding resistance to chloramphenicol, sulfonamides, trimethoprim, and tetracyclines were detected in 261 [64.7%; mainly *cat*B3 (46.2%)], 280 [69.5%; *sul*1 (36.2%) and *sul*2 (40.9%)], 252 [62%; mainly *dfrA14* (42.7%)], and 106 [26.3%, mainly *tet*A (23.1%)] isolates, respectively. Plasmid-mediated quinolone resistance *qnr*-like determinants were detected in 202 (50.1%) isolates, with *qnr*B1-like (37.7%) as the most frequent.

Seven isolates contained colistin ARGs; *mcr-9* in four CP-Kpn and two CP-Eco and *mcr-1* in one CP-Eco. However, all six isolates harboring *mcr-9* were susceptible to colistin, as previously described (Macesic et al., 2021). There were 36 colistin-resistant CP-Kpn isolates, all of them showing amino acid substitution in both proteins of the PhoQ/PhoP regulatory system: D1509G and H406Y in PhoQ protein and R114A and L26Q in PhoP (Elias et al., 2021). Additional MgrB mutations were detected in four isolates: stop codon in L4 (3 ST512/KPC-3 isolates) and W47C (1 ST307/OXA-48 isolates).

The most prevalent ARGs associated with high-risk CP-Kpn clones are detailed in Table 2. *bla*_SHV–28_ allele was detected in 96.3% of ST307 but only in 21.1% of other clones. Additionally, the association of the *rmtF* and *arr* genes with ST147 was detected in 52.8 and 66.7%, respectively, compared with 0 and 6.2% in other clones.

CP-Kpn isolates belonged to 46 capsular polysaccharide loci (K-loci); 24 K-loci contained more than one isolate, and six included 78.3% of isolates, namely, KL102 (80), KL24 (75), KL107 (56), KL64 (39), KL112 (29), and KL27 (17). High correlations between K-loci and STs were observed: all KL102 isolates were ST307, all KL112 isolates were ST15, 92% of KL24 isolates were ST11, 87% of KL64 isolates were ST147, and 94.5% of KL107 isolates were ST512-258.

The yersiniabactin-encoding locus (*ybt*) was detected in 43% of CP-Kpn isolates, particularly ST11 (40.2%), ST147 (15.9%), and ST15 (14.6%). Nine different *ybt* lineages were identified, with *ybt*10, associated with ICEKp4 and ST11, being the most frequent (58.5%; Supplementary Figure 1). Colibactin (*clb*) and aerobactin (*iuc*) loci were detected in two isolates each (Supplementary Figure 1). One ST147/NDM-1 isolate contained the *rmpA2* gene associated with a hypermucoid/hypervirulent phenotype.

### Characterization of Plasmids Harboring Carbapenemases Genes

The plasmidID mapping tool reconstructed three IncFIB plasmids carrying *bla*_KPC_ genes, all of them highly similar to NZ_CP027056 of ∼90,000 bp (average identity > 95 and 99.9% coverage). In these three plasmids, *bla*_KPC_ genes were carried in the transposon Tn*4401b*, which was modified by the insertion of Tn*5403*, similar to that previously described (Rada et al., 2020).

In the ST147/NDM-1 isolate, *bla*_NDM–1_ was carried by an IncFIB plasmid of ∼105,000 bp (highly similar to NZ_CM008884, average identity > 95% and 99.82% coverage). The largest genetic environment constructed for this gene was 5,943 bp, with the sequence *gro*L-*gro*S-*nag*A-*trp*F-*ble*-*bla*_NDM–1_-(trun)ISAba, which is similar to the previously described pNDM-11_IncFIB_KPN_Spain (Pérez-Vázquez et al., 2019a).

The plasmids detected carrying *bla*_OXA–48_ (ST307/OXA-48 isolate) and *bla*_VIM–1_ (ST11/VIM-1 isolate) were IncL. The first one was highly similar to NZ_CP023251 (∼63,000 bp, average identity > 95% and 100% coverage) and the other to NZ_CP023419 (∼70,000 bp, average identity > 95% and 100% coverage). The *bla*_OXA–48_ gene was located in a Tn*1999* in which *lysR* and *bla*_OXA–48_ were flanked by two copies of *IS1999* (Mairi et al., 2018). The *bla*_VIM–1_ gene was located in the class 1 integron In*624*, showing the sequence *Int*1-*bla*_VIM–1_-*aacA4*-*dfrB1*-*aadA1*-*catB2*-*qacEdelta1*-*sul*1 (Villa et al., 2014).

## Discussion

We conducted a comprehensive analysis using WGS in CP-Kpn and CP-Eco isolates in Spain, revealing an overall cumulative incidence of 0.05 per 100 admitted patients and the wide interregional dissemination of high-risk clones. The study’s strengths include representation of all Spanish provinces, application of population denominators for prevalence and incidence calculations, and the possibility of establishing evolutionary trends compared with previous studies using a similar methodology conducted by the same research team (Miró et al., 2013; Oteo et al., 2015; Grundmann et al., 2017). Although the use of only 10 strains per hospital might be a limitation, this approach aligns with ECDC strategies (Grundmann et al., 2017), avoiding the overrepresentation of geographical regions/clones observed in other studies.

The overall incidence of CP-Kpn and CP-Eco increased by 25%, from 0.04 cases per 100 patients in 2014 (Grundmann et al., 2017), with 13 provinces reporting incidences at least 2.5-fold higher in this study than the general one found in 2014. Compared with the 2.5% CP-Kpn prevalence reported here, the prevalences detected in 2009 and 2013 in Spain were significantly lower, at 0.2% (Miró et al., 2013) and 1.7% (Oteo et al., 2015), respectively. Increased prevalence was accompanied by a wide geographical spread, with strains detected in 92% of the 50 Spanish provinces and the presence of seven “high-risk clones” in at least three provinces. In a recent Italian study (Di Pilato et al., 2021), 80% of 30 participating hospitals reported CP-Kpn cases. The large difference in CP-Kpn prevalences between provinces detected in this study (four provinces had prevalences > 10%, Supplementary Table 2) highlights the importance of designing a study with representation from all geographical regions and with a design that minimizes the possible overrepresentation of a specific clone or a region due to the existence of an outbreak. In this sense, our study provides a realistic and representative view of the Spanish situation, minimizing possible biases.

Our results confirm previous reports of the preponderance of OXA-48 carbapenemase in Spain (detected in 82% of Spanish provinces; Oteo et al., 2015; Grundmann et al., 2017; Vázquez-Ucha et al., 2021). The *bla*_OXA–505_ gene (OXA-48 family, GenBank number: NG_049783) was detected in a carbapenem non-susceptible isolate with a positive colorimetric lacking other genes encoding carbapenemases, so it was included in the study despite the absence of published evidence on its carbapenemase activity.

New to this study was the observation of significant epidemiological evolution among CP-Kpn strains, from the predominance of the OXA-48–producing ST11 and ST405 (Oteo et al., 2015) to the emergence of ST307/OXA-48 and the dispersion of ST512/KPC (detected in seven provinces in Southern Spain). Compared with a multicenter study conducted in 2013 (Oteo et al., 2015), our study shows a significant increase in all high-risk clones, except ST11/OXA-48, which decreased in frequency from 24.1 to 16.4%, and ST15, which remained stable (Table 2). The frequency of ST307 increased from 1.4% in 2013 (Oteo et al., 2015) to 16.4%, making it the predominant clone with a wide geographical distribution. Other recent studies also have identified that ST307 is an emerging clone worldwide (Wyres et al., 2019; Di Pilato et al., 2021), and the hyperepidemic clonal complex ST258/ST512 is widely predominant in Italy and Greece (David et al., 2019; Di Pilato et al., 2021). This epidemiological shift is associated with the increased CP-Kpn population diversity, from an SDI of 10.6 in 2013 (Oteo et al., 2015) to 12.7 in this study. Regarding the NDM-producers, the main high-risk *K. pneumoniae* clone ST147/NDM-1 detected in this study (Table 2) was the cause of one of the great NDM-1-producing *K. pneumoniae* outbreaks previously described in Spain (Pérez-Vázquez et al., 2019b).

CPE bacteremia is associated with high mortality (Tamma et al., 2017), mainly associated with delays in adequate treatment (Gutiérrez-Gutiérrez et al., 2017). This study showed a significantly higher prevalence of CP-Kpn in bacteremia than in the total infections considered altogether (more than double) or urinary tract infections (more than fourfold), with ST512/KPC and ST15/OXA-48 causing 40% of CP-Kpn–induced bacteremia. Accurate and timely diagnosis could be critical in providing effective care.

Regarding the level of resistance to carbapenem antibiotics as the main target of carbapenemases, it should be noted that the susceptibility profile of meropenem and/or imipenem susceptibility with ertapenem resistance was frequently detected in this study. This profile was mainly due to the high prevalence of OXA-48 isolates, although it can also be found in VIM-1 producers (Oteo et al., 2015; Vázquez-Ucha et al., 2021).

Vaborbactam and relebactam do not inhibit metallo-β-lactamases; however, differences in susceptibility were observed between imipenem/imipenem-relebactam and meropenem/meropenem-vaborbactam in this collection (Table 1B). These discrepancies were due to the different cutoff points established by EUCAST in the case of meropenem-vaborbactam and to the five isolates that had MICs of 4 mg/L and 2 mg/L for imipenem and imipenem/relebactam, respectively.

New antibiotics (cefiderocol, plazomicin, meropenem/vaborbactam, and imipenem/relebactam) have significantly improved the treatment options for CPE infections (Doi, 2019). In our study, all CP-Kpn isolates showed > 75% susceptibility to these antibiotics. In accordance with previous studies (Doi, 2019), plazomicin was not active against ST147 isolates carrying *rmtF*1 (OXA-48 or NDM-1 producers), and meropenem/vaborbactam and imipenem/relebactam activities were higher in KPC-producers compared with OXA-48 and metallo-β-lactamases producers. As previously described (Yamano, 2019), cefiderocol showed good activity *in vitro* against CP-Kpn, irrespective of the carbapenemase types. The seven KPC-23-producing isolates were resistant (CMI > 16 mg/L) to ceftazidime/avibactam. This carbapenemase was previously associated with a decrease in ceftazidime/avibactam susceptibility (Galani et al., 2019). Overall susceptibility to colistin and meropenem decreased from 95.5 to 81.3%, respectively, in 2013 (Oteo et al., 2015) to 90.5% and 52.3% in this study, due primarily to the rise in KPC and NDM. The rate of colistin resistance was lower than previously reported, associated with KPC-producers (Di Pilato et al., 2021) but was consistent with the rate reported in a recent Spanish study (Vázquez-Ucha et al., 2021).

The increasing worldwide dispersion of carbapenemases is due to a mixed spread: i) clonal, with the existence of high-risk clones predominant in the carbapenemase-producing *K. pneumoniae* population, and ii) polyclonal, with the spread of conjugative epidemic plasmids capable of carrying the different carbapenemases genes (Villa et al., 2014; Mairi et al., 2018; Pérez-Vázquez et al., 2019a; Kraftova et al., 2021).

Our study elucidates the epidemiology of CP-Kpn and CP-Eco in Spain using WGS in 403 clinical isolates, representing a first step toward the integration of WGS in CPE surveillance in Spain, compliant with the high-priority recommendations of the ECDC (ECDC, 2019). Our findings will aid in the development of the Network of Laboratories for the Surveillance of Resistant Microorganisms (RedLabRA; Cañada-García et al., 2021). The data generated by this study may serve as a reliable benchmark for CP-Kpn status in Spain in the year before the COVID-19 pandemic, facilitating implementation of control measures.

## Data Availability Statement

The datasets presented in this study can be found in online repositories. The names of the repository/repositories and accession number(s) can be found in the article/Supplementary Material.

## Ethics Statement

The studies involving human participants were reviewed and approved by the Spanish Agency for Medicines and Health Products (code JOI-AVI-2019-01) and the Research Ethics Committee of the University Hospitals Virgen Macarena and Virgen del Rocio (Sevilla, Spain). Written informed consent from the participants’ legal guardian/next of kin was not required to participate in this study in accordance with the national legislation and the institutional requirements.

## GEMARA/GEIRAS-SEIMC/REIPI CARB-ES-19 Study Group

Members of the GEMARA/GEIRAS-SEIMC/REIPI CARB-ES-19 Collaborating Group are as follows:

Mariela Martínez Ramírez (H. Universitario de Guadalajara, Guadalajara); Pilar Zamarrón (Hospital Virgen de la Salud, Toledo); Miriam Albert Hernández (Hospital Virgen de la Concha Complejo Asistencial de Zamora, Zamora); M. Pilar Ortega Lafont (Complejo Asistencial Universitario de Burgos, Burgos); Emilia Cercenado (H. General Universitario Gregorio Marañón, CIBERES Madrid); Cristobal del Rosario and Jose Luis Perez Arellano (Hospital Universitario Insular Materno Infantil de Gran Canaria, Las Palmas); María Lecuona (Hospital Universitario de Canarias, La Laguna, Sta. Cruz Tenerife); Luis López-Urrutia Lorente (H. Universitario Río Hortega, Valladolid); José Leiva and José Luis del Pozo (Clínica Universidad de Navarra, Navarra); Salvador Giner and Juan Frasquet (H. Universitario La Fe de Valencia, Valencia); Lidia Garcia Agudo and Soledad Illescas (H General Universitario de Ciudad Real, Ciudad Real); Pedro de la Iglesia (Hospital de Cabueñes, Asturias); Rosario Sánchez Benito (Hospital San Pedro de Alcántara, Cáceres); Eugenio Garduño (Hospital Universitario Badajoz, Badajoz); M^a^ Isabel Fernández Natal and Marta Arias (Complejo Asistencial Universitario de León, León); Marta Lamata Subero (Fundación Hospital de Calahorra Megalab, La Rioja); Mar Olga Pérez Moreno (H. Verge de la Cinta de Tortosa, Tarragona); Ana Isabel López-Calleja (H. Universitario Miguel Servet, Zaragoza); Luis Torres Sopena (H. San Jorge de Huesca, Huesca); José Manuel Azcona (H. San Pedro, La Rioja); Alba Belles and Mercè García González (H. Universitario Arnau de Vilanova, Lleida); Miriam Valverde Troya and Begoña Palop (Hospital Regional Universitario de Málaga, Málaga); Fernando García Garrote (Hospital Universitario Lucus Augusti, Lugo); Jose Luis Barrios Andrés and Leyre López Soria (Hospital Universitario Cruces, Vizcaya); Adelina Gimeno (H. General Universitario de Alicante, Alicante); Susana Sabater (Hospital General de Castellón, Castellón); Ester Clapés Sanchez (Hospital Dr. Josep Trueta de Girona, Girona); Jennifer Villa (H. Universitario 12 de Octubre, Madrid); Nuria Iglesias Nuñez and Rafael Sánchez Arroyo (Hospital Nuestra Señora de Sonsoles Ávila); Inmaculada García García (Complejo Hospitalario de Salamanca, Salamanca); Susana Hernando (Real Hospital General de Segovia, Segovia); Cristina Seral and Javier Castillo (H. Clínico Universitario Lozano Blesa, Zaragoza); Eva Riquelme Bravo, Caridad Sainz de Baranda, and Oscar Esparcia Rodríguez (C. Hospitalario Universitario de Albacete, Albacete); Jorge Gaitán, María Huertas (Hospital General La Mancha Centro Alcazar, Ciudad Real); M.^a^ José Rodríguez Escudero (Hospital General Virgen de La Luz, Cuenca); Carmen Aldea and Nerea Sanchez (Complejo Asistencial de Soria, Soria); Antonio Casabella Pernas (Hospital Universitari Parc Taulí, Institut d’Investigació i Innovació Parc Taulí I3PT, Universitat Autònoma de Barcelona, Sabadell, Barcelona); M^a^ Dolores Quesada (H. Germans Trias I Pujol, Badalona Barcelona); Maria Pilar Chocarro and Francisco Javier Ramos (Hospital Obispo Polanco, Teruel); Carmina Martí Sala (H. General de Granollers, Barcelona); Laura Mora, Encarnación Clavijo (H. Virgen de la Victoria, Málaga); Natalia Chueca and Federico García (H. Clínico San Cecilio, Granada); José Gutierrez Fernández (Hospital Virgen de las Nieves, Granada); Juan Manuel Sánchez Hospital de Jérez (Jerez de la Frontera, Cádiz); Fátima Galán Sánchez (H. Puerta del Mar, Cádiz); Carmen Liébana and Carolina Roldán (Hospital Universitario de Jaén, Jaén); M^a^ Isabel Cabeza (Hospital de Poniente, Almería); José María Saavedra (Hospital Juan Ramón Jimenez Huelva); M^a^ Teresa Cabezas Fernández (Complejo Hospitalario de Torrecárdenas, Almería); Lucía Martínez Lamas and Sonia Rey Cao (Complejo Hospitalario Universitario de Vigo, Pontevedra); M^a^ Isabel Paz Vidal (Complejo Hospitalario de Orense, Orense); Raquel Elisa Rodríguez Tarazona (Hospital Santos Reyes, Aranda de Duero, Burgos); Amparo Coira Nieto and M^a^ Luisa Pérez del Molino Bernal (Hospital Clínico Universitario de Santiago de Compostela A Coruña); María Gomáriz Díaz (H. Universitario de Donostia, Guipuzcoa); Matxalen Vidal-García and Jose Luis Díaz de Tuesta (Hospital Universitario de Basurto, IIS Biocruces, Bizkaia); Moises García Bravo and Almudena Tinajas (H. General Río Carrión, Palencia); Andrés Canut Blasco and M^a^ Luz Albina Cordón Rodriguez (H. Universitario de Álava, Álava); Nieves Gonzalo Jiménez (H. General Universitario de Elche, Alicante); Genoveva Yagüe Guirao (Hospital Virgen de la Arrixaca de Murcia, Murcia); Fe Tubau Quintano (Hospital Universitario de Bellvitge, Barcelona); Carmen Aspiroz (H. Royo Villanova Zaragoza); Nuria Prim (H. del Mar, Barcelona); Jesús Rodríguez-Baño (H. Virgen Macarena, CIBERINFEC, Seville).

## Author Contributions

JO-I and MP-V conceived and designed the study. JC-G, ZM, ÁP, ZP-B, AO, JV, RC, GB, JG-L, FN, LM-M, GR-C, and JO-I coordinated the study. JC-G, PS-C, MD-V, MC, DG, MG, IG-A, NL, XM, CP, AR, BA, and GEMARA-SEIMC/REIPI CARB-ES-19 Study Group performed the experiments. ZM, JC, MP-V, and JO-I wrote the manuscript. All authors have read, edited, and approved the final manuscript.

## Conflict of Interest

The authors declare that the research was conducted in the absence of any commercial or financial relationships that could be construed as a potential conflict of interest.

## Publisher’s Note

All claims expressed in this article are solely those of the authors and do not necessarily represent those of their affiliated organizations, or those of the publisher, the editors and the reviewers. Any product that may be evaluated in this article, or claim that may be made by its manufacturer, is not guaranteed or endorsed by the publisher.

## Footnotes

1
https://microreact.org/project/vXbqq3eF5WXY5qZe9HBUJL-carb-es-2019

2
http://quast.sourceforge.net/

3
https://iTOL.embl.de/

4
http://www.genomicepidemiology.org/?

5
https://github.com/katholt/Kleborate

6
https://github.com/BU-ISCIII/plasmidID

## Supplementary Material

Supplementary Figure 1 Distribution of yersiniabactin lineages and K-types in high-risk carbapenemase-producing *Klebsiella pneumoniae* clones.
