# Corrigendum: CARB-ES-19 multicenter study of carbapenemase-producing *Klebsiella pneumoniae* and *Escherichia coli* from all Spanish provinces reveals interregional spread of high-risk clones such as ST307/OXA-48 and ST512/KPC-3

**DOI:** 10.3389/fmicb.2023.1331657

**Published:** 2023-11-28

**Authors:** Javier E. Cañada-García, Zaira Moure, Pedro J. Sola-Campoy, Mercedes Delgado-Valverde, María E. Cano, Desirèe Gijón, Mónica González, Irene Gracia-Ahufinger, Nieves Larrosa, Xavier Mulet, Cristina Pitart, Alba Rivera, Germán Bou, Jorge Calvo, Rafael Cantón, Juan José González-López, Luis Martínez-Martínez, Ferran Navarro, Antonio Oliver, Zaira R. Palacios-Baena, Álvaro Pascual, Guillermo Ruiz-Carrascoso, Jordi Vila, Belén Aracil, María Pérez-Vázquez, Jesús Oteo-Iglesias, The GEMARA/GEIRAS-SEIMC/REIPI CARB-ES-19 Study Group

**Affiliations:** ^1^Laboratorio de Referencia e Investigación en Resistencia a Antibióticos e Infecciones Relacionadas con la Asistencia Sanitaria, Centro Nacional de Microbiología, Instituto de Salud Carlos III, Madrid, Spain; ^2^Escuela Internacional de Doctorado, Ciencias Biomédicas y Salud Pública - IMIENS (UNED), Madrid, Spain; ^3^Unidad de Enfermedades Infecciosas y Microbiología, Hospital Universitario Virgen Macarena, Instituto de Biomedicina de Sevilla (Hospital Universitario Virgen Macarena/CSIC/Universidad de Sevilla), Seville, Spain; ^4^CIBER de Enfermedades Infecciosas (CIBERINFEC), REIPI, Instituto de Salud Carlos III, Madrid, Spain; ^5^Servicio de Microbiología, Hospital Universitario Marqués de Valdecilla, IDIVAL, Santander, Spain; ^6^Servicio de Microbiología, Hospital Universitario Ramón y Cajal, Instituto Ramón y Cajal de Investigación Sanitaria (IRYCIS), Madrid, Spain; ^7^Servicio Microbiología, Hospital Universitario A Coruña, Instituto Investigación Biomédica A Coruña (INIBIC), A Coruña, Spain; ^8^Microbiology Unit, Reina Sofia University Hospital, Maimonides Biomedical Research Institute of Cordoba (IMIBIC), Córdoba, Spain; ^9^Departament de Genetica i Microbiologia, Servei de Microbiologia, Hospital Universitari Vall d'Hebron, Universitat Autònoma de Barcelona, Barcelona, Spain; ^10^Servicio de Microbiología, Hospital Universitario Son Espases, Instituto de investigación sanitaria Illes Balears (IdISBa), Palma de Mallorca, Spain; ^11^Servicio de Microbiología, Hospital Clínic de Barcelona, ISGlobal Barcelona Institute for Global Health, Barcelona, Spain; ^12^Microbiology Department, Hospital de la Santa Creu i Sant Pau, Universitat Autónoma de Barcelona (UAB), Sant Pau Biomedical Research Institute (IIB Sant Pau), Barcelona, Spain; ^13^Department of Agricultural Chemistry, Soil Science and Microbiology, University of Córdoba, Córdoba, Spain; ^14^Departamento de Microbiología, Universidad de Sevilla, Seville, Spain; ^15^Servicio de Microbiología Clínica, Hospital Universitario La Paz (IdiPAz), Madrid, Spain

**Keywords:** CARB-ES-19 study, carbapenemases, whole genome sequencing, *Klebsiella pneumoniae*, high-risk clones

In the published article, there was an error regarding the affiliation for **Javier E. Cañada-García**. As well as having affiliation **1**, he should also have “^**2**^**Escuela Internacional de Doctorado, Ciencias Biomédicas y Salud Pública - IMIENS (UNED), Madrid, Spain**.” This affiliation has been added to the list of author affiliations.

The authors apologize for this error and state that this does not change the scientific conclusions of the article in any way. The original article has been updated.

