# Supplementary material for: CARB-ES-19 Multicenter Study of Carbapenemase-Producing Klebsiella pneumoniae and Escherichia coli From All Spanish Provinces Reveals Interregional Spread of High-Risk Clones Such as ST307/OXA-48 and ST512/KPC-3
Source: Front Microbiol. 2022 Jun 30;13:918362. doi: 10.3389/fmicb.2022.918362 (PMC9279682; doi:10.3389/fmicb.2022.918362)
Supplement: Supplementary file 1 [file Table_1.pdf]

Table S1. Primers used for carbapenemases genes amplification.

Target	Primer	Sequence (5′ to 3 ′, as synthesized)	Expected amplicon size (bp)
OXA-48	OXA-48-F	TCACAGGGCGTAGTTGTGCT	126
	OXA-48-R	GAGGGCGATCAAGCTATTGG	
VIM	VIM-F	TTTGACCGCCTCTATCATGG	125
	VIM-R	CGGACAATGAGACCATTGGA	
NDM	NDM-F	AGGACAAGATGGGCGGTATG	124
	NDM-R	GCGAAAGTCAGGCTGGTTG	
KPC	KPC-F	GTCGGAGACAAAACCGGAAC	102
	KPC-R	GGTGTAGACGGCCAACACAA	
IMP	IMP-F	TTCCATAGCGACAGCACRGG	88
	IMP-R	GGGCCRGGRTARAAACTTC	

The PCR program consisted of an initial denaturation step at 95°C for 10 min, followed by 45 cycles of DNA denaturation at 95°C for 15s, primer annealing at 51°C for 30s, and primer extension at 72°C for 15s. After the last cycle, a final extension step at 72°C for 10 min was added
