# Supplementary material for: CARB-ES-19 Multicenter Study of Carbapenemase-Producing Klebsiella pneumoniae and Escherichia coli From All Spanish Provinces Reveals Interregional Spread of High-Risk Clones Such as ST307/OXA-48 and ST512/KPC-3
Source: Front Microbiol. 2022 Jun 30;13:918362. doi: 10.3389/fmicb.2022.918362 (PMC9279682; doi:10.3389/fmicb.2022.918362)
Supplement: Supplementary file 2 [file Table_2.pdf]

Table S2. Distribution by Spanish provinces of the participating hospitals, number of cases, incidence and prevalence of CPE.

Province	Number of hospitals	CP* *K. pneumoniae*	CP* *E.coli*	CP* *K. pneumoniae prevalence by province*	CP* *E.coli prevalence by province*	CPE** Cumulative incidence	CPE** Incidence density
Álava	1	2	0	0.76	0	0.02	0.04
Albacete	1	1	1	0.29	0.05	0.01	0.02
Alicante	2	8	0	0.97	0	0.06	0.11
Almería	2	4	0	0.82	0	0.03	0.04
Asturias	1	6	4	2.05	0.22	0.09	0.14
Ávila	1	4	0	3.74	0	0.05	0.09
Badajoz	1	3	0	1.84	0	0.32	0.22
Baleares	1	0	2	0	0.24	0.03	0.04
Barcelona	7	42	9	3.06	0.15	0.03	0.05
Burgos	2	3	0	1.54	0	0.02	0.03
Cáceres	1	10	0	4.22	0	0.18	0.29
Cádiz	2	16	0	4.94	0	0.06	0.08
Cantabria	1	10	0	13.70	0	0.23	0.53
Castellón	1	0	0	0	0	0	0
Ciudad Real	2	16	0	5.14	0	0.06	0.08
Córdoba	1	10	0	8.26	0	0.09	0.14
Cuenca	1	1	0	1.79	0	0.03	0.05
Girona	1	9	1	2.38	0.06	0.13	0.32
Gran Canaria	1	9	1	7.44	0.31	0.33	0.34
Granada	2	10	0	3.48	0	0.05	0.10
Guadalajara	1	10	0	7.87	0	0.34	0.58
Guipuzcoa	1	2	0	0.42	0	0.01	0.03
Huelva	1	2	1	0.73	0.07	0.05	0.08
Huesca	1	0	0	0	0	0	0
Jaén	1	5	0	5.43	0	0.13	0.14
La Coruña	2	18	0	3.06	0	0.04	0.05
La Rioja	2	2	0	0.63	0	0.03	0.05
León	1	8	0	4.19	0	0.09	0.13
Lérida	1	1	0	0.20	0	0.02	0.04
Lugo	1	10	0	5.78	0	0.16	0.19
Madrid	4	34	4	5.21	0.17	0.20	0.30
Malaga	2	14	0	3.85	0	0.15	0.21
Murcia	1	0	0	0	0	0	0
Navarra	1	1	0	2.13	0	0.06	0.07
Orense	1	8	2	3.32	0.14	0.01	0.02
Palencia	1	10	0	11.76	0	0.16	0.27
Pontevedra	1	10	0	5.62	0	0.16	0.29
Salamanca	1	0	0	0	0	0	0
Segovia	1	9	1	10.11	0.16	0.09	0.16
Sevilla	1	3	0	4.69	0	0.03	0.05
Soria	1	1	0	1.03	0	0.04	0.05
St Cruz Tenerife	1	10	0	14.08	0	0.31	0.32
Tarragona	1	2	0	1.18	0	0.03	0.07
Teruel	1	1	0	1.20	0	0	0
Toledo	1	9	0	1.56	0	0.04	0.07
Valencia	1	10	0	3.50	0	0.10	0.16
Valladolid	1	8	0	3.86	0	0.03	0.05
Vizcaya	2	16	0	1.92	0	0.05	0.09
Zamora	1	6	0	4.00	0	0.04	0.06
Zaragoza	3	3	0	0.35	0	0.01	0.01
**Total**	71	377	26	2.50	0.04	0.05	0.08

***CP**: carbapenemase-producing
****CPE**: carbapenemase-producing Enterobacterales (including only *Klebsiella pneumoniae* and *Escherichia coli*)
