# Supplementary material for: CARB-ES-19 Multicenter Study of Carbapenemase-Producing Klebsiella pneumoniae and Escherichia coli From All Spanish Provinces Reveals Interregional Spread of High-Risk Clones Such as ST307/OXA-48 and ST512/KPC-3
Source: Front Microbiol. 2022 Jun 30;13:918362. doi: 10.3389/fmicb.2022.918362 (PMC9279682; doi:10.3389/fmicb.2022.918362)
Supplement: Supplementary file 3 [file Table_3.pdf]

Table S3. Antibiotic susceptibility of 26 carbapenemase-producing *Escherichia coli* isolates as determined by the microdilution method and antibiotic gradient strips (antibiotics with *) according to EUCAST clinical breakpoints.

**Antibiotics**	**S (%)**	**R (%)**	**MIC_{50}***	**MIC_{90}***	**Range***
Meropenem/Vaborbactam*	100	0	0.25	2	0.03 - 4
Plazomicin*	100	0	1	2	0.06 - 2
Cefiderocol*	96.2	3.8	0.03	0.25	≤0.015 - 4
Colistin	92.3	7.7	1	1	0.5 - 8
Meropenem	88.5	0	1	3	0.25 - 8
Imipenem/Relebactam*	84.6	15.4	1	4	0.25 - 8
Tigecycline	84.6	15.4	0.5	1	≤0.25 - 1
Amikacin	84.6	15.4	≤4	16	≤4 - 16
Ceftazidime/Avibactam	76.9	23.1	≤0.5	>16	≤0.5 - >16
Imipenem	69.2	7.7	2	4	≤0.5 - 16
Gentamicin	65.4	34.6	1	>8	≤0.5 - >8
Tobramycin	57.7	42.3	≤1	>8	≤1 - >8
Cefepime*	50	46.2	2.5	32	0.12 - >256
Aztreonam	50	38.5	1.5	>32	≤0.5 - >32
Trimethoprim/Sulfamethoxazole	46.2	53.8	>8	>8	≤1 - >8
Ceftazidime	42.3	53.8	16	>16	≤0.5 - >16
Ceftolozane/Tazobactam	42.3	57.7	12	>32	≤0.5 - >32
Cefotaxime	23.1	61.5	>8	>8	≤0.5 - >8
Ciprofloxacin	19.2	76.9	>2	>2	≤0.06 - >2
Ertapenem	15.4	84.6	2	>2	0.5 - >2
Piperacillin/Tazobactam	0	100	>32	>32	>32 - >32

S: susceptible. R: resistant. MIC: minimum inhibitory concentrations.

* Expressed in mg/L.
