# Supplementary material for: CARB-ES-19 Multicenter Study of Carbapenemase-Producing Klebsiella pneumoniae and Escherichia coli From All Spanish Provinces Reveals Interregional Spread of High-Risk Clones Such as ST307/OXA-48 and ST512/KPC-3
Source: Front Microbiol. 2022 Jun 30;13:918362. doi: 10.3389/fmicb.2022.918362 (PMC9279682; doi:10.3389/fmicb.2022.918362)
Supplement: Supplementary file 4 [file Table_4.pdf]

Table S4. Main characteristics of six main clusters identified by a maximum-likelihood phylogenetic tree using 92,608 high-quality SNPs (Figure 2).

Clusters	Nº isolates (%)	ST	Predominant carbapenemase genes (n)	Nº provinces	SNPs of difference within the cluster (range)
Cluster 1	81 (21.8)	307	OXA-48 (61), KPC-3 (14)	18	53 (0-259)
Cluster 2	70 (18.9)	11	OXA-48 (66)	23	193 (0-2087)
Cluster 3	42 (11.3)	15	OXA-48 (35)	15	131 (0-356)
Cluster 4	52 (13.8)	258 (4), 512 (48)	KPC-3 (45), KPC-23 (7)	8	20 (0-37)
Cluster 5	35 (9.4)	147	OXA-48 (22), NDM-1 (7)	12	40 (0-162)
Cluster 6	15 (4)	392	OXA-48 (16)	7	42 (0-63)
