# Supplementary figures and images for: CARB-ES-19 Multicenter Study of Carbapenemase-Producing Klebsiella pneumoniae and Escherichia coli From All Spanish Provinces Reveals Interregional Spread of High-Risk Clones Such as ST307/OXA-48 and ST512/KPC-3
Source: Front Microbiol. 2022 Jun 30;13:918362. doi: 10.3389/fmicb.2022.918362 (PMC9279682; doi:10.3389/fmicb.2022.918362)

A)

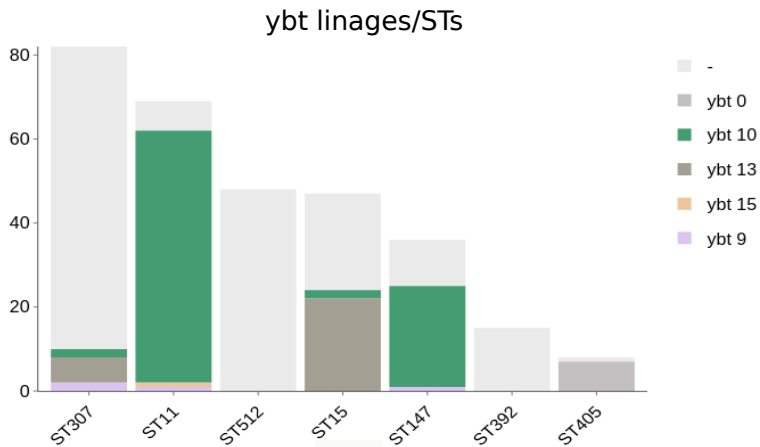

B)

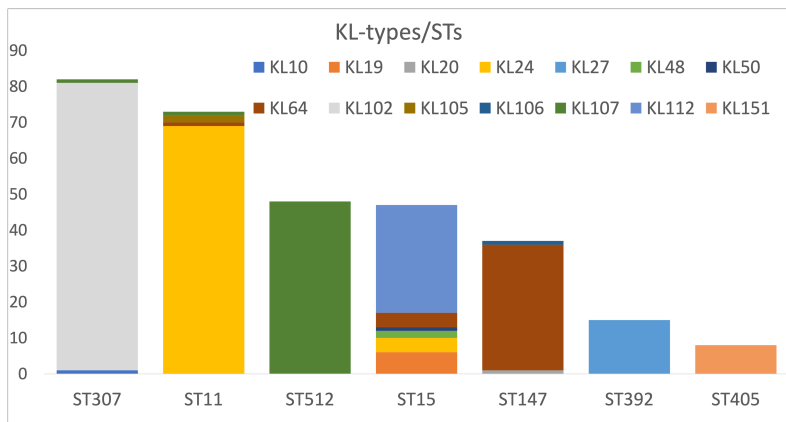

Supplement: Supplementary Figure 1 — Distribution of yersiniabactin lineages and K-types in high-risk carbapenemase-producing Klebsiella pneumoniae clones. [file Image_1.pdf]
